# Author Correction: Microbiome and epigenetic variation in wild fish with low genetic diversity

**DOI:** 10.1038/s41467-024-49904-8

**Published:** 2024-07-03

**Authors:** Ishrat Z. Anka, Tamsyn M. Uren Webster, Waldir M. Berbel-Filho, Matthew Hitchings, Benjamin Overland, Sarah Weller, Carlos Garcia de Leaniz, Sofia Consuegra

**Affiliations:** 1 Department of Biosciences, Centre for Sustainable Aquatic Research, Swansea University, Swansea, Wales SA2 8PP UK; 2 Department of Aquaculture, Chattogram Veterinary and Animal Sciences University, Chattogram, 4225 Bangladesh; 3 Department of Biology, University of Oklahoma, Norman, OK 73019 USA; 4 Institute of Life Science, Swansea University, Swansea, Wales SA2 8PP UK; 5 Marine Research Centre (CIM-UVIGO), Universidade de Vigo, Vigo, Spain; 6 Grupo de Biotecnología Acuática, Departamento de Biotecnología y Acuicultura, Instituto de Investigacións Mariñas, IIM-CSIC Vigo, Spain; 7 Present Address: Department of Biology, University of West Florida, Pensacola, FL USA

**Keywords:** Ecological genetics, Genetic variation, Evolutionary genetics

Correction to: *Nature Communications* 10.1038/s41467-024-49162-8, published online 03 June 2024

The original version of this Article contained an error in Fig. 2, in which panel A displayed data from the wrong populations of the study species. The correct version of Fig. 2 is:


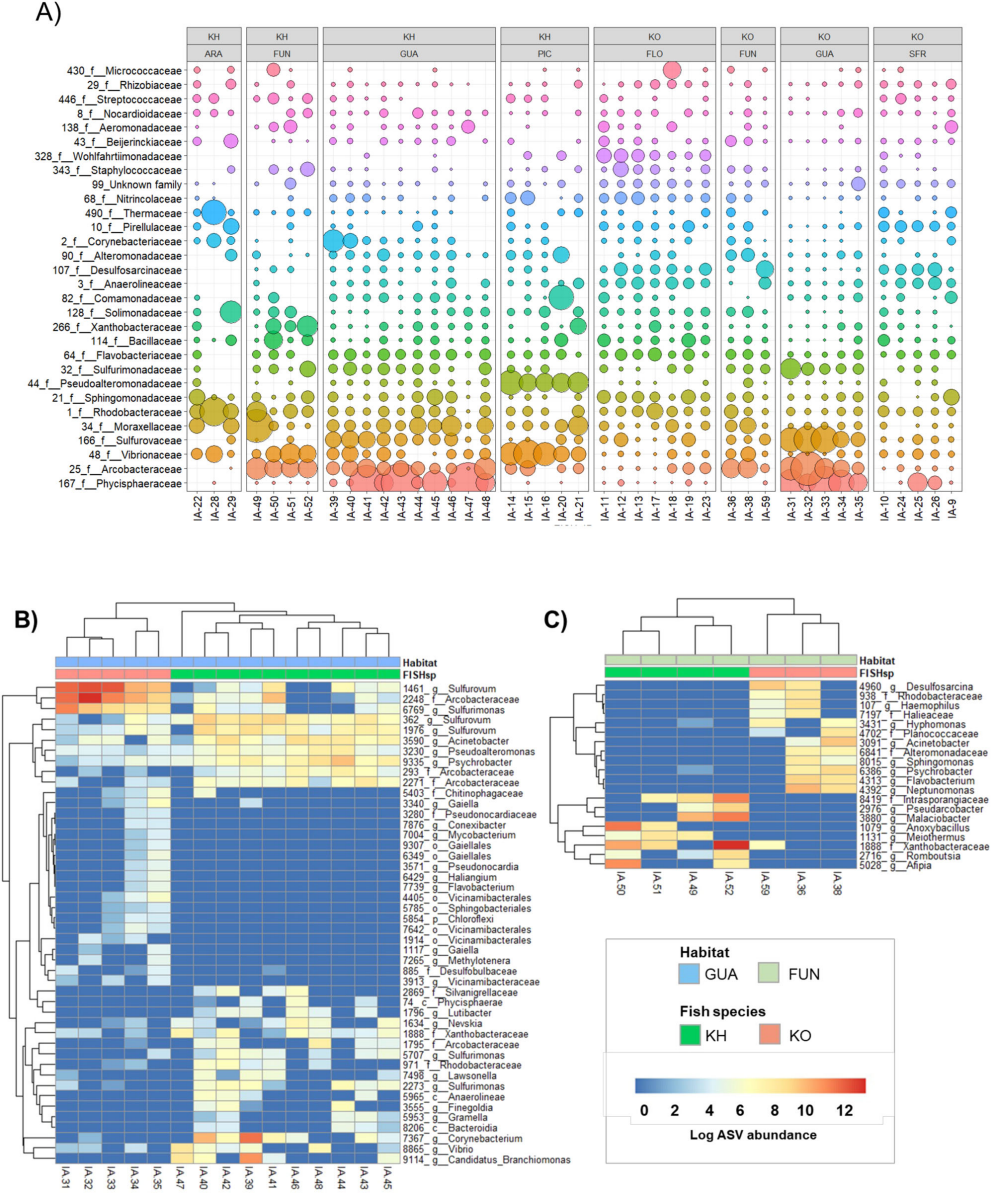


which replaces the previous incorrect version:
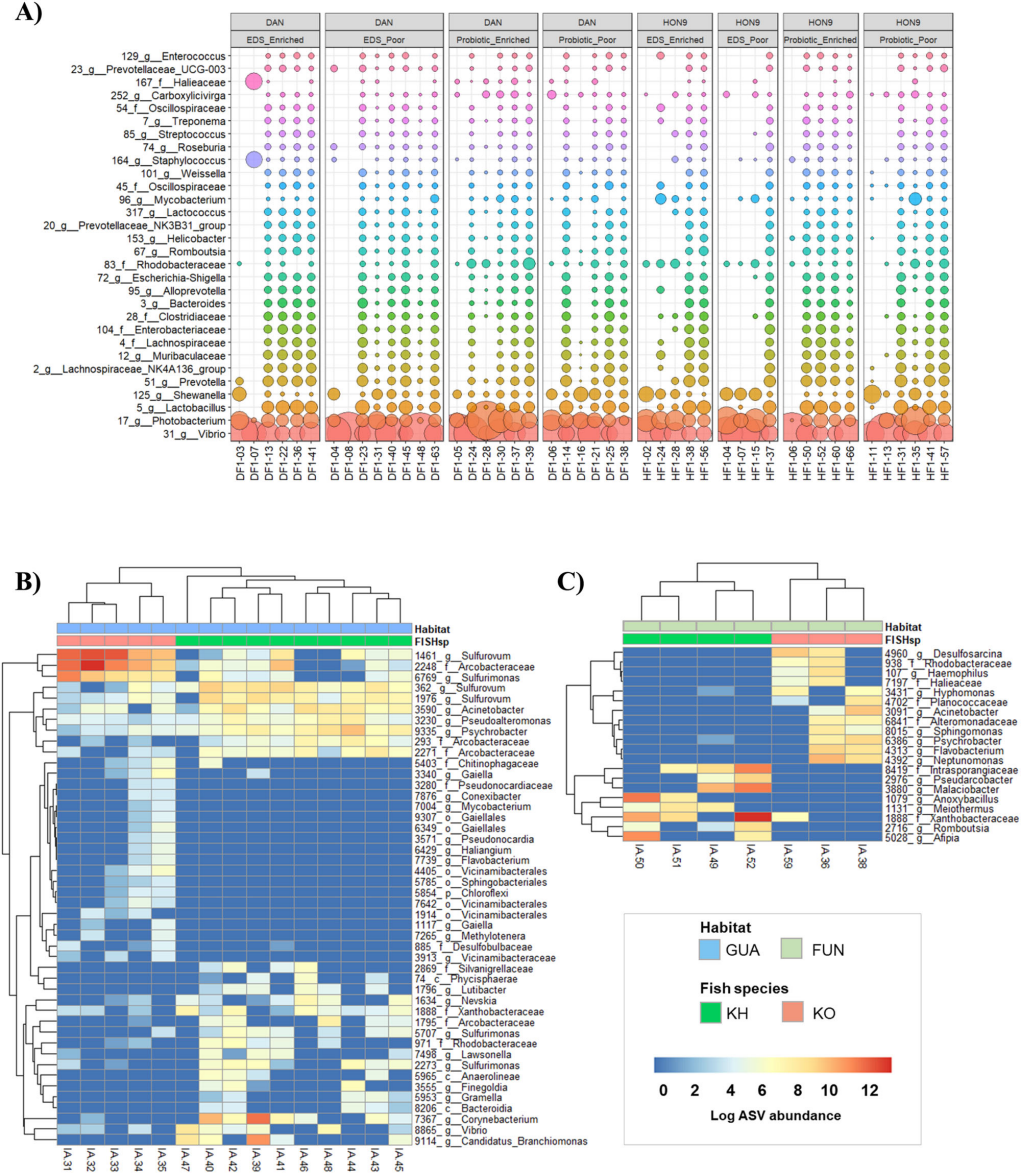


This has been corrected in both the PDF and HTML versions of the Article.

