# Supplementary material for: Microbiome and epigenetic variation in wild fish with low genetic diversity
Source: Nat Commun. 2024 Jun 3;15:4725. doi: 10.1038/s41467-024-49162-8 (PMC11148108; doi:10.1038/s41467-024-49162-8)

Supplementary Information for:

Microbiome and epigenetic variation in wild fish with low genetic diversity

Ishrat Z. Anka[1,2], Tamsyn M. Uren Webster[1], Waldir M. Berbel-Filho[3], Matthew Hitchings[4], Benjamin Overland[1], Sarah Weller[1], Carlos Garcia de Leaniz[1], Sofia Consuegra[1*]

[1] Department of Biosciences, Centre for Sustainable Aquatic Research, Swansea University, Swansea, Wales SA2 8PP, UK

[2]Department of Aquaculture, Chattogram Veterinary and Animal Sciences University, Chattogram 4225, Bangladesh

[3]Department of Biology, University of Oklahoma, Norman, OK 73019, USA

[4]Institute of Life Science, Swansea University, Swansea, Wales SA2 8PP, UK

*Corresponding author: Sofia Consuegra, s.consuegra@swansea.ac.uk

Supplementary Figure 1. Predicted community metagenomic profiling. Displaying the 20 most abundant MetaCyc pathways represented across all communities identified using PICRUSt2 v2.5.2.

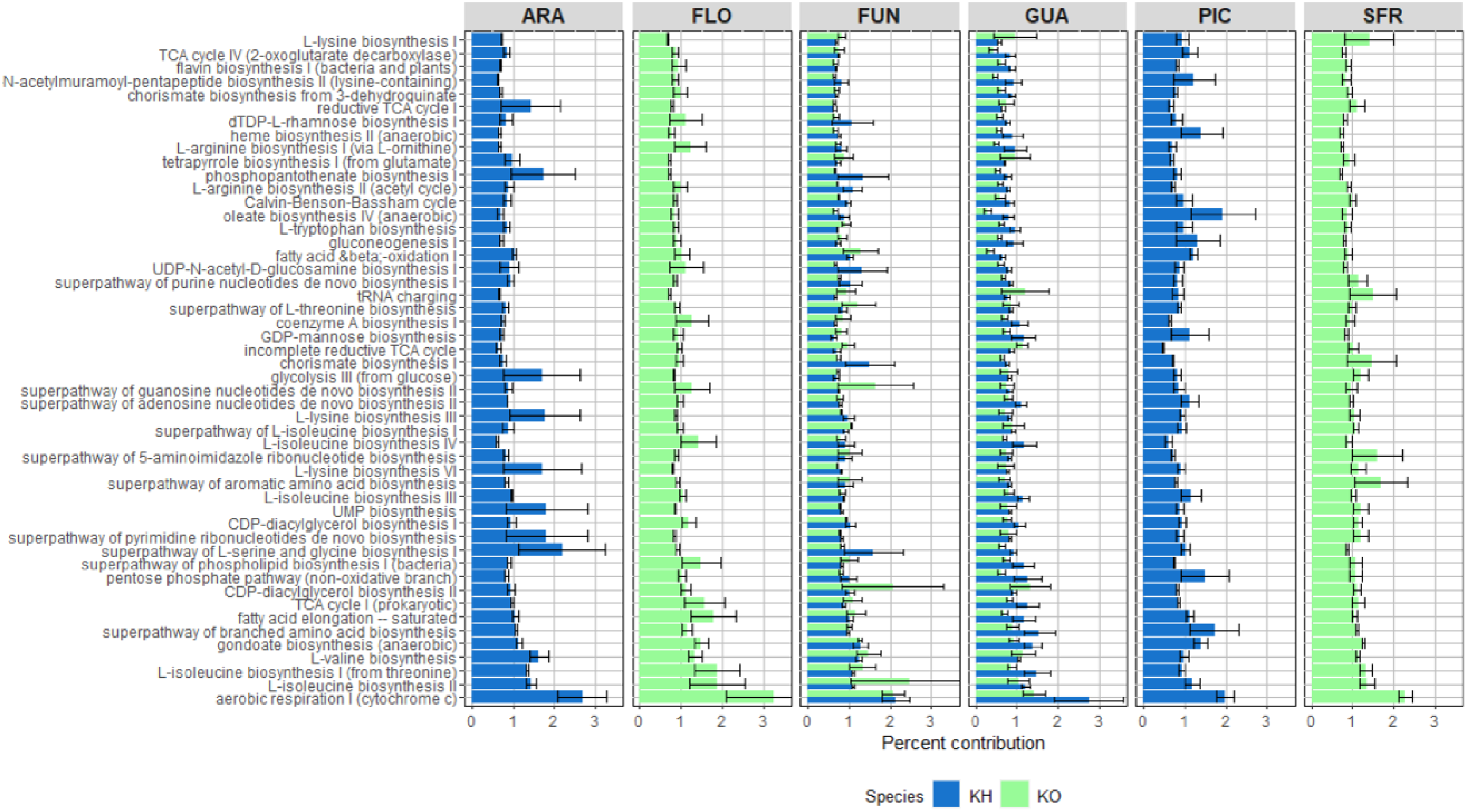

Supplementary Figure 2. Median and predicted CIs for non-parametric bootstrapped and linear models of microbiome alpha diversity. Chao1, Faith_pd, Shannon and Simpson_e = alpha diversity estimates. Single data points are shown in grey. Source data is provided as source data file.

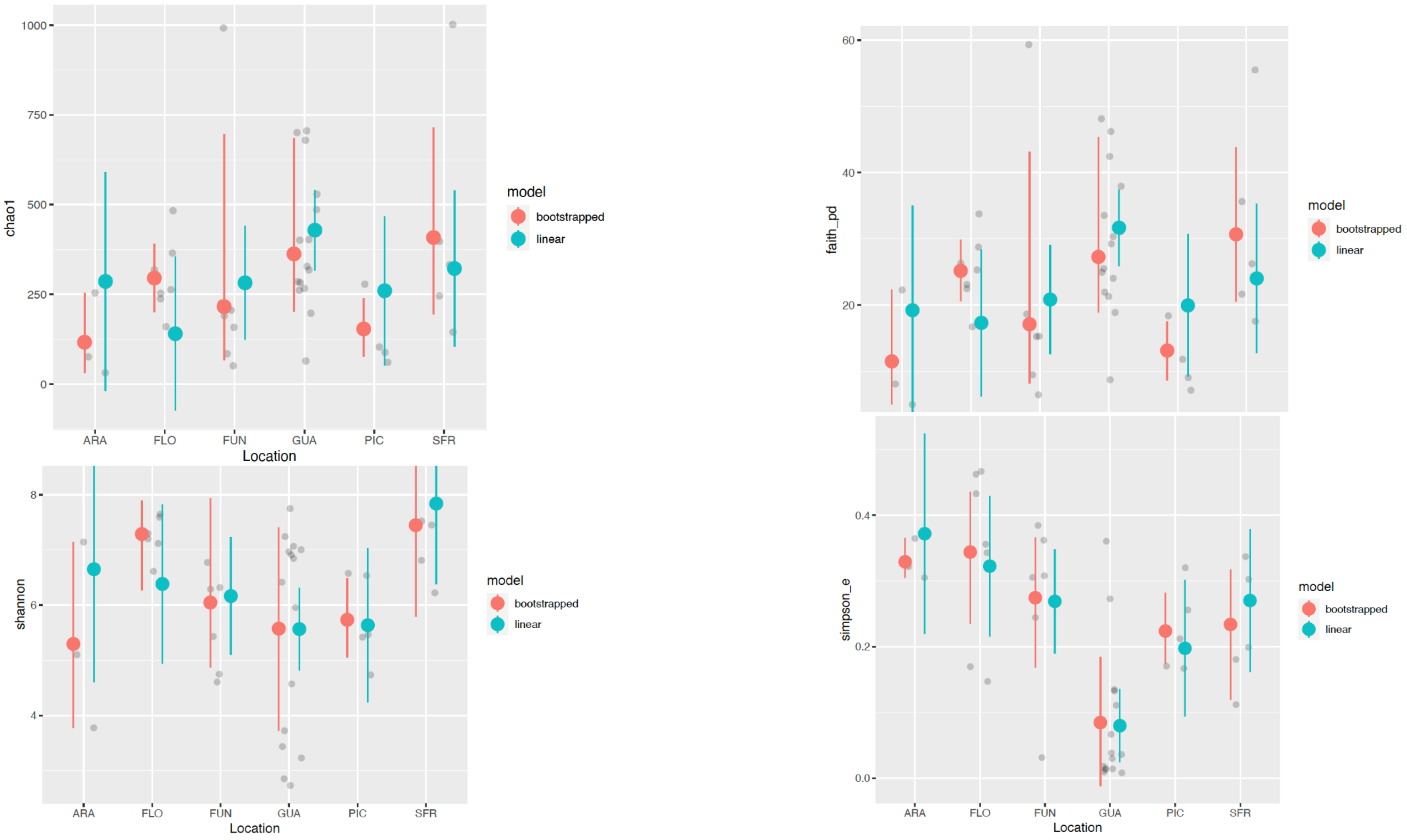

Supplementary Figure 3. Microbiome alpha diversity estimates for *Kryptolebias hermaphroditus* and *K. ocellatus* in sympatry or allopatry. Key: *Kryptolebias hermaphroditus* (KH n=22) and *K. ocellatus* (KO n=20); sympatry (SYMP) or allopatry (ALLO). Data was analysed using linear models: lm(alpha diversity ~ Species + Habitat share). Chao1 diversity was significantly influenced by species and sympatry (Species: F= 10.819 P= 0.002; Sympatry: F= 4.575 P=0.038), and so was Faith PD (Species: F= 17.118 P= 0.002; Sympatry: F= 5.780 P=0.02), unlike Shannon diversity (Species: F= 3.486 P= 0.069; Sympatry: F= 2.177 P=0.148) or Simpson's evenness which was influenced only by sympatry (Species: F= 0.641 P= 0.428; Sympatry: F= 11.638 P=0.002). Source data is provided as source data file.

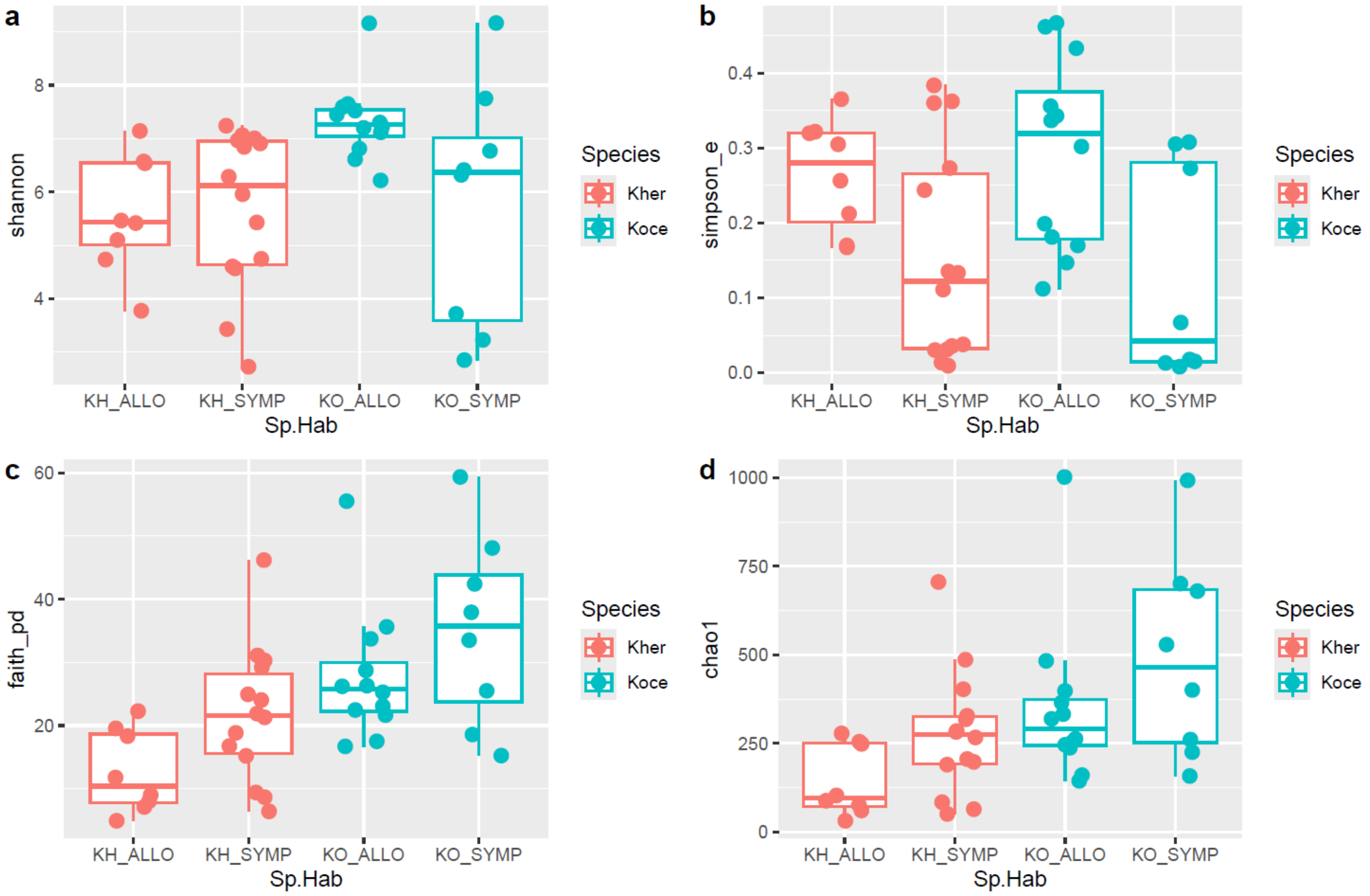

Supplementary Figure 4. Relationship between individual heterozygosity and sympatry. Heterozygosity estimates (H.indiv) for *Kryptolebias hermaphroditus* (KH n=14) and *K. ocellatus* (KO n=14) in sympatry and allopatry (SYMP and ALLO). Data was analysed using linear models: lm(H_indiv ~ Species + Habitat share). Species (F= 186.571 P<0.001) and sympatry (shared or non-shared location; F=28.101 P<0.001) both influenced individual heterozygosity Source data is provided as source data file.

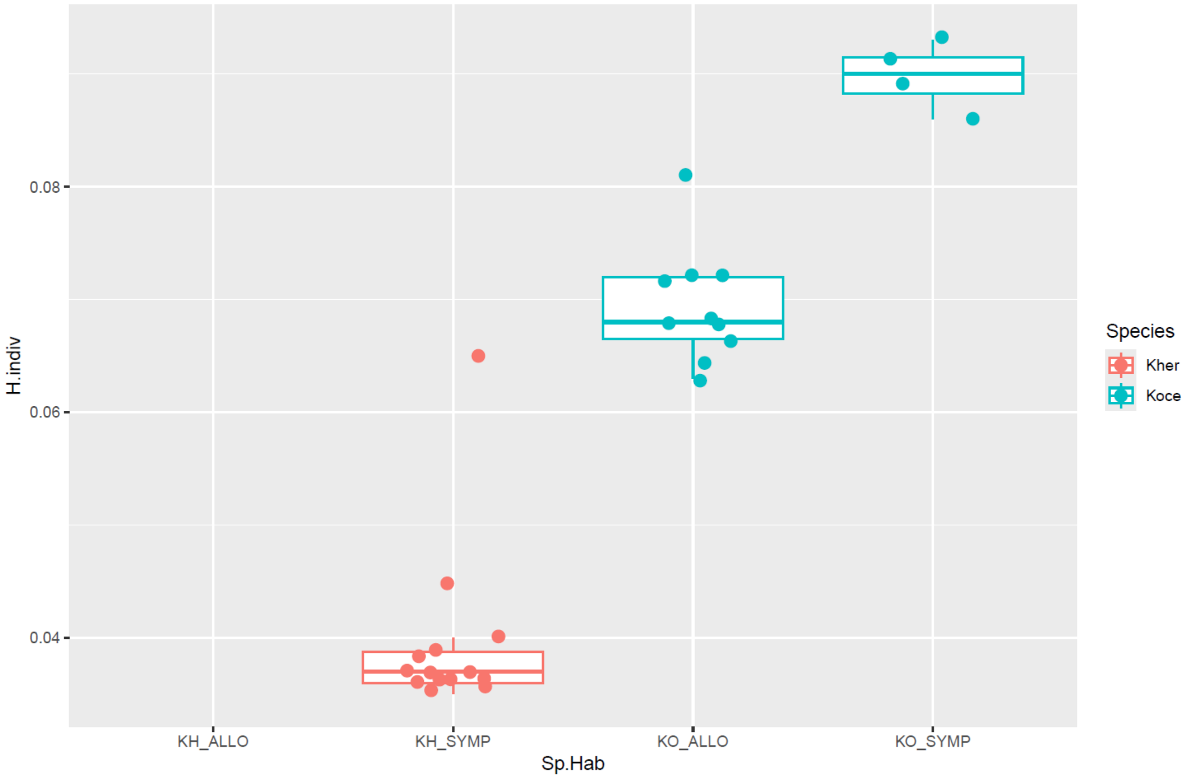

Supplementary Figure 5. Relationships between microbiome and genetic distances. Relationship between genetic (Euclidean distance) and microbiome dissimilarity (Bray-Curtis and weighted Unifract distance) in *Kryptolebias hermaphroditus* (n=14) and *K. ocellatus* (n=14). Mantel tests using 10,000 permutations: Euclidean genetic distance and weighted Unifrac microbiome dissimilarity Mantel R= 0.155 P=0.04; Euclidean genetic distance and Bray-Curtis microbiome dissimilarity Mantel R= -0.134 P=0.108. Source data is provided as source data file.

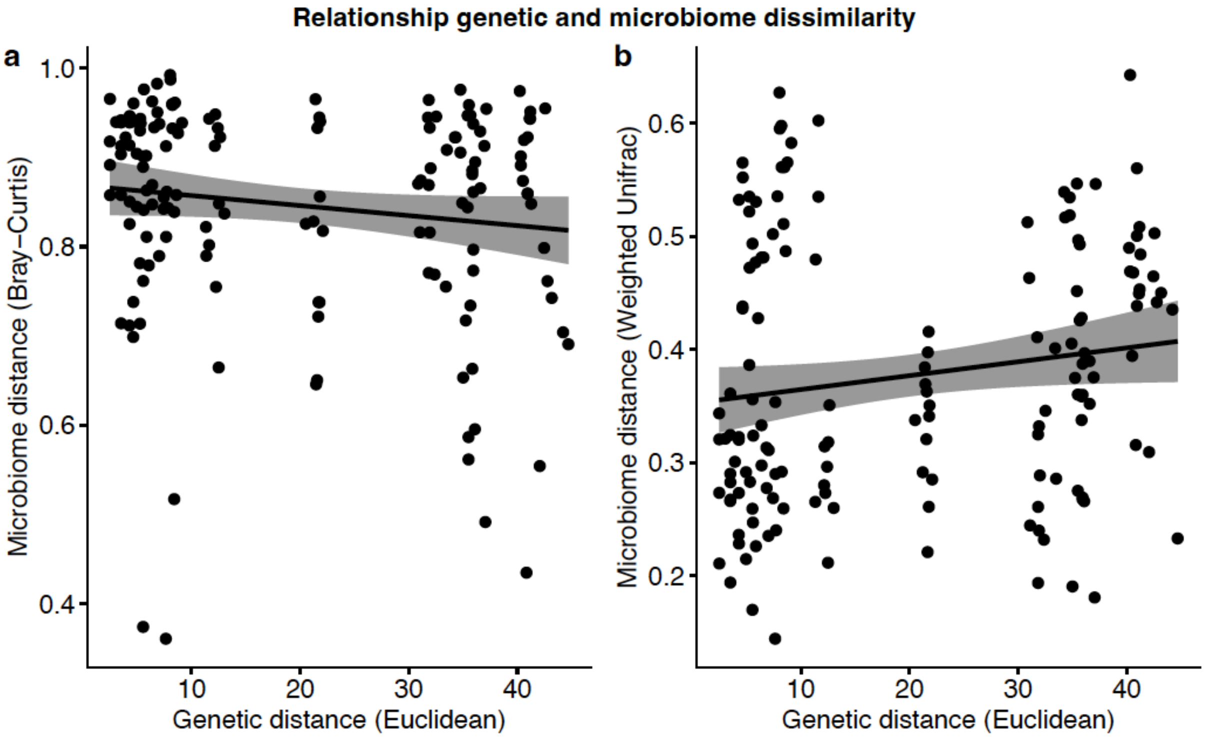

Supplementary Figure 6. Fluctuating asymmetry differences between species and relationship with DNA methylation. (a-c) Fluctuating asymmetry in ocellus area, eye diameter and both combined (total asymmetry) in *Kryptolebias hermaphroditus* (n=10) and *K. ocellatus* (n=11). Total asymmetry differences between species were tested with a Welch two-sample t=test, t = -2.0886, df = 19, p-value = 0.05 and (d) Relationship between total asymmetry and epigenetic diversity (DNA methylation coefficient of variation, CV) for both species (n=14). Probability for Pearson correlation coefficient is two-sided. Source data is provided as source data file.

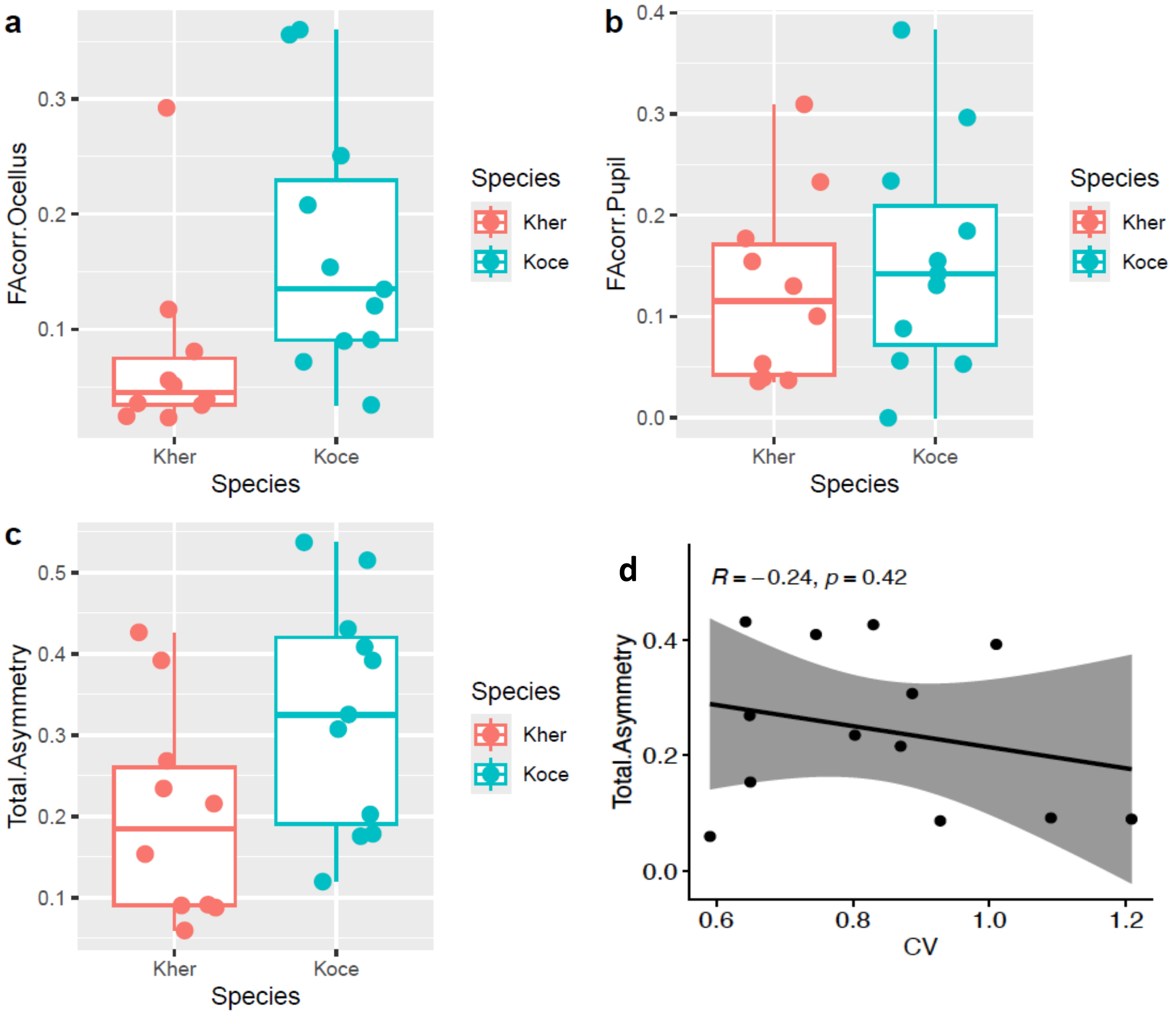

Supplement: Supplementary file 1 — Supplementary Information [file 41467_2024_49162_MOESM1_ESM.pdf]
