# Supplementary material for: Microbiome and epigenetic variation in wild fish with low genetic diversity
Source: Nat Commun. 2024 Jun 3;15:4725. doi: 10.1038/s41467-024-49162-8 (PMC11148108; doi:10.1038/s41467-024-49162-8)
Supplement: Supplementary file 3 — Description of Additional Supplementary Files [file 41467_2024_49162_MOESM3_ESM.pdf]

Description of Additional Supplementary Files

File Name: Supplementary Data 1

Description: Data for individual fish (Sample) including: species, sampling location, standard length (mm), alpha diversity metrics (chao1, shannon, simpson_eveness and faith_phylogenetic diversity), individual heterozygosity (H-indiv) based on SNPs, and coefficient of variation of DNA methylation based on counts per million of 64,152 methylated sites (CV) measurements for K. ocellatus (Koce) and K. hermaphroditus (Kher).

File Name: Supplementary Data 2

Description: Analysis of indicator Amplicon Sequence Variants (ASVs) associated with the locations (GUA and FUN) where *Kryptolebias hermaphroditus* (KH) and *K. ocellatus* (KO) were sampled in sympatry. The Association statistic (IndVal) is a measure of the strength of the association between each ASVs and the respective group (species or location). The statistical significance of this relationship is tested using a permutation test, two-sided.

File Name: Supplementary Data 3

Description: Analysis of indicator Amplicon Sequence Variants (ASVs) associated with *Kryptolebias hermaphroditus* (KH) and *K. ocellatus* (KO) sampled in sympatry in GUA and FUN locations. The Association statistic (IndVal) is a measure of the strength of the association between each ASVs and the respective group (species or location). The statistical significance of this relationship is tested using a permutation test, two-sided.

File Name: Supplementary Data 4

Description: Snapshot of water parameters at the time of sampling each one of the locations.

File Name: Supplementary Data 5

Description: Results from non-parametric regression analyses of microbiome alpha diversity analyses, based on 1,000 bootstrapping replicates for all linear models: A) Species and location as predictors (N=42), B) Species, location and individual heterozygosity as predictors, species removed due to colinearity (N=28, see Table 1). Probabilities based on the 1,000 replicates are two tailed. Mean and median probabilities for all bootstrapped models are also provided.

File Name: Supplementary Data 6

Description: Individual identification, sampling location and pairwise distances (Bray-Curtis and Unifrac for microbiome, Euclidean for genetic distance based on SNPs) for fish (14 Kryptolebias hermaphroditus and 14 K. ocelatus) living in sympatry FUN, GUA) and allopatry (FLO, SFR).

File Name: Supplementary Data 7

Description: Individual identification, sampling location and pairwise distances Bray-Curtis for DNA methylation and microbiome, Euclidean for genetic distance based on SNPs for 18 fish (14 Kryotolebias hermaphroditus and 4 K. ocelatus) living in sympatry (FUN and GUA locations).

File Name: Supplementary Data 8

Description: Repeated measurements of fluctuating asymmetry (1st and 2nd measurements) and corrected values (FA1 and FA2 corrected following Torcs et al 2016) for three morphometric traits (ocellus area, distance from eye to snout and eye diameter) of *Kryptolebias hermaphroditus* and *K. ocellatus* in sympatric locations (FUN and GUA).

File Name: Supplementary Data 9

Description: Results of two-way ANOVA analysis of fluctuating asymmetry including side (left or right) and individual as factors, and F-ratio tests of significance, following Palmer (1994) and Tocts et al. (2016). Probabilities are two sided.
