# Supplementary material for: Microbiome and epigenetic variation in wild fish with low genetic diversity
Source: Nat Commun. 2024 Jun 3;15:4725. doi: 10.1038/s41467-024-49162-8 (PMC11148108; doi:10.1038/s41467-024-49162-8)
Supplement: Supplementary file 5 — Reporting Summary [file 41467_2024_49162_MOESM5_ESM.pdf]

nature portfolio

Corresponding author(s): Sofia Consuegra
Last updated by author(s): Apr 8, 2024

Reporting Summary

Nature Portfolio wishes to improve the reproducibility of the work that we publish. This form provides structure for consistency and transparency in reporting. For further information on Nature Portfolio policies, see our Editorial Policies and the Editorial Policy Checklist.

Statistics

For all statistical analyses, confirm that the following items are present in the figure legend, table legend, main text, or Methods section.

n/a	Confirmed	
☐	☒	The exact sample size (*n*) for each experimental group/condition, given as a discrete number and unit of measurement
☐	☒	A statement on whether measurements were taken from distinct samples or whether the same sample was measured repeatedly
☐	☒	The statistical test(s) used AND whether they are one- or two-sided<br>*Only common tests should be described solely by name; describe more complex techniques in the Methods section.*
☐	☒	A description of all covariates tested
☐	☒	A description of any assumptions or corrections, such as tests of normality and adjustment for multiple comparisons
☐	☒	A full description of the statistical parameters including central tendency (e.g. means) or other basic estimates (e.g. regression coefficient) AND variation (e.g. standard deviation) or associated estimates of uncertainty (e.g. confidence intervals)
☒	☐	For null hypothesis testing, the test statistic (e.g. *F*, *t*, *r*) with confidence intervals, effect sizes, degrees of freedom and *P* value noted<br>*Give P values as exact values whenever suitable.*
☒	☐	For Bayesian analysis, information on the choice of priors and Markov chain Monte Carlo settings
☐	☒	For hierarchical and complex designs, identification of the appropriate level for tests and full reporting of outcomes
☐	☒	Estimates of effect sizes (e.g. Cohen's *d*, Pearson's *r*), indicating how they were calculated

Our web collection on statistics for biologists contains articles on many of the points above.

Software and code

Policy information about availability of computer code

Data collection	Sequence analysis was performed as described in81 using Qiime2 (version: qiime2-2022.2). Primer trimming, denoising and removal of chimeras was done with DADA2. Classification was then performed using the Silva reference taxonomy (v138;Quast et al. 2012). Predicted community metagenomic profiling was performed using PICRUSt2 v2.5.2. Whole community enzyme classification (EC) number abundances were calculated and subsequently used to infer MetaCyc pathway abundances using MinPath.
Data analysis	Statistical differences in ASV abundance were examined using DeSeq2. ASV relative abundance was visualised using Pheatmap (Kolde, 2015), based on Euclidean distance clustering. We also used the function multipatt in IndicSpecies to identify ASVs significantly associated with species and location. Differential analysis of predicted functional pathway representation was performed using ALDEx2 v1.30.0, using the glm tool with a Holm-Bonferroni FWER correction .<br>All statistical analysis were carried out in R v4.2.2 90. Linear models were done using the function lm and model comparison was carried out by examining changes in AIC and using the anova command. Multivariate analysis of variance of microbiome data was performed using adonis. Linear mixed models were carried out using the functions lmer and drop1in lmerTest to identify the best model for analyses of microbiome and genetic diversity. The performance and ecodist packages were used to test for model assumptions and carry out multiple regression analyses of distance matrices.

For manuscripts utilizing custom algorithms or software that are central to the research but not yet described in published literature, software must be made available to editors and reviewers. We strongly encourage code deposition in a community repository (e.g. GitHub). See the Nature Portfolio guidelines for submitting code & software for further information.

Data

Policy information about availability of data

All manuscripts must include a data availability statement. This statement should provide the following information, where applicable:

- Accession codes, unique identifiers, or web links for publicly available datasets
- A description of any restrictions on data availability
- For clinical datasets or third party data, please ensure that the statement adheres to our policy

Microbiome sequences have been submitted to the European Nucleotide Archive (ENA) under accession number PRJEB61741: https://www.ebi.ac.uk/ena/browser/text-search?query=PRJEB61741
FastaQC files for the GBS library for the SNPs and DNA methylation data can be accessed at NCBI accession number PRJNA563625: https://www.ncbi.nlm.nih.gov/bioproject/PRJNA563625/
Scripts for DNA methylation and SNP bioinformatics processing are available at: https://github.com/waldirmbf/BerbelFilho_etal_KryptolebiasHybridisation/tree/master/1.ProcessingSequencingFiles/1.2.EpigeneticAnalysis .

Research involving human participants, their data, or biological material

Policy information about studies with human participants or human data. See also policy information about sex, gender (identity/presentation), and sexual orientation and race, ethnicity and racism.

Reporting on sex and gender	NA
Reporting on race, ethnicity, or other socially relevant groupings	NA
Population characteristics	NA
Recruitment	NA
Ethics oversight	NA

Note that full information on the approval of the study protocol must also be provided in the manuscript.

Field-specific reporting

Please select the one below that is the best fit for your research. If you are not sure, read the appropriate sections before making your selection.

☐ Life sciences ☐ Behavioural & social sciences ☒ Ecological, evolutionary & environmental sciences

For a reference copy of the document with all sections, see nature.com/documents/nr-reporting-summary-flat.pdf

Ecological, evolutionary & environmental sciences study design

All studies must disclose on these points even when the disclosure is negative.

Study description	Skin swabs were collected from Kryptolebias hermaphroditus (N= 22; Standard length mean=28.27 SD= 4.90) and Kryptolebias ocellatus (N= 20; Standard length mean =28.80 SD= 7.17), two closely related mangrove killifish, from six sites in south and southeast Brazil, two sites where both species coexisted in sympatry (Guaratiba and Fundão; GUA and FUN), two sites only inhabited by K. ocellatus (Florianópolis and São Francisco do Sul; FLOR and SFR) and two sites only inhabited by K. hermaphroditus (Picinguaba and Aracruz; PIC and ARA. For microbiome analysis, DNA was extracted and amplified using 16S rRNA primers and libraries (and blanks) were sequenced in a MiSeq Illumina platform (300 bp, paired end). SNP data collected in a previous study was analysed from 14 individuals of each species (28 in total) for which both SNP and microbiome data was available. We analysed the influence of host genetics (pairwise distance and heterozygosity based on SNPs) on microbiome alpha and beta diversity, using as well sampling location (proxy for environmental influence), size of the fish and species as precictors. Epigenetic data (DNA methylation) from the same previous study was available for 18 fish (14 K. hermaphroditus and 4 K. ocellatus) from the sympatric locations (GUA and FUN) and we analysed the influence of microbiome alpha and beta diversity, genetic pairwise distance and heterozygosity, fish size, sampling location and species on epigenetic pairwise distance and methylation coefficient of variation (as an indicator of individual epigenetic variation). Fluctuating asymmetry analyses were carried out for all the fish DNA methylation data.
Research sample	Two closely related species of mangrove killifishes were studied, Kryptolebias hermaphroditus and Kryptolebias ocellatus were sampled from six sites in south and southeast Brazil, two sites where both species coexisted in sympatry, two sites only inhabited by K. ocellatus and SFR) and two sites only inhabited by K. hermaphroditus, to assess the environmental influcence. The species were chosen because of their contracting mating systems that result in different gradients of genetic diversity. K. hermaphroditus is one of the only two known self-fertilising hermaphrodites in vertebrate and its populations consists mainly of self-fertilising hermaphrodites with males at very low frequencies45. Outcrossing rarely occurs between K. hermaprhoditus males and hermaphrodites, which are

	typically inbred with very high homozygosity levels. In contrast, K. ocellatus populations consist of males and hermaphrodites in approximately equal ratio and only reproduce via outcrossing. K. ocellatus populations display more genetic diversity than K. hermaphroditus.
Sampling strategy	The aim was a sample size of 30/species/location, based on previous studies where this was found enough to identify statistically significant population genetic differences (based on microsatellites). However, as sampling of these species takes place using hand nets and it is very challenging due to their small size and the nature of the mangrove environment, the number of fish captured per location varied between 6 and 31.
Data collection	Waldir Berbel-Filho collected the field samples and took the fish swabs. Ishrat Anka and Tamsyn Uren Webster carried out the DNA extractions of the swabs and the libraries and did the Bioinformatics analyses. Matt Hitchings run the libraries and collected the raw microbiome data. Ben Overland and Sarah Weller carried out fish measurements and FA analyses.
Timing and spatial scale	Fish sampling took place between July and August 2017. SNP and epigenetic data collection took place between January and June 2018 approximately. DNA extraction from swabs and microbiome data collection took place between March and June 2022. FA analyses took place in November-December 2023.
Data exclusions	There were no data exclusions.
Reproducibility	This study did not involve manipulation and no attempt has been done to repeat the experiment as it is based on the analyses of samples collected in the field. Analyses of genetic diversity corroborated previous observations of higher heterozygosity of the self-fertilising species compared to the outcrossing one.
Randomization	Groups consisted of species and locations, these were chosen based on whether both species were coexisting or just one of them was inhabiting the location. The rationale was to control for the influence of the environment in microbiome composition. Location and species were included as predictors in all analyses. Individuals were included as random effects in pairwise comparisons, to take into account the fact that the same individual was compared several times.
Blinding	Blinding was not relevant in this case as the data had to be assigned to the different groups for the analysis.

Did the study involve field work? ☒ Yes ☐ No

Field work, collection and transport

Field conditions	Sampling took place between July and August 2017. Weather was dry, water temperature oscillated between 18C and 22C and dissolved oxygen between 6.7 and 80.4 ppm.
Location	FUN Fundão mangrove, Rio de Janeiro, RJ 22°52′2.50″S 43°13′27.50″W<br>GUA Piracão mangrove, Guaratiba, RJ 23°0′1.90″S 43°34′51.50″W<br>PIC Fazenda River, Picinguaba, SP 23°22′01.0″S 044°50′13.4″W<br>SFR Linguado channel, São Francisco do Sul, SC 26°22′0.02″S 48°39′58.40″W<br>FLO Rio Ratones estuary, Florianópolis, SC 27°28′3.84″S 48°29′33.76″W
Access & import/export	Sampling complied with all Brazilian laws and was carried out under license ICMBio/SISBIO 57145-1/2017, which included exportation permit.
Disturbance	Sampling was carried out with hand nets which causes minimal impact on the natural mangrove environment .

Reporting for specific materials, systems and methods

We require information from authors about some types of materials, experimental systems and methods used in many studies. Here, indicate whether each material, system or method listed is relevant to your study. If you are not sure if a list item applies to your research, read the appropriate section before selecting a response.

Materials & experimental systems

n/a	Involved in the study	
☒	☐	Antibodies
☒	☐	Eukaryotic cell lines
☒	☐	Palaeontology and archaeology
☐	☒	Animals and other organisms
☒	☐	Clinical data
☒	☐	Dual use research of concern
☒	☐	Plants

Methods

n/a	Involved in the study	
☒	☐	ChIP-seq
☒	☐	Flow cytometry
☒	☐	MRI-based neuroimaging

Animals and other research organisms

Policy information about studies involving animals; ARRIVE guidelines recommended for reporting animal research, and Sex and Gender in Research

Laboratory animals	No laboratory animals were used in the study
Wild animals	Swabs were taken from wild fish for microbiome analyses. Fish were euthanised in the field with an overdose of anaesthesic as for Home Office procedures.
Reporting on sex	Sex (male or hermaphrodite) was inferred by body and fin coloration patterns, which are reliably used for sex differentiation in mangrove killifish species. In K. ocellatus, males were identified by a black spot on the dorsal part of the caudal fin. In K. hermaphroditus, males were identified by the presence of a broad black margin along the whole caudal fin, bordered by a broad submarginal white zone. Sex was not used as a covariate due to the small proportion of males included (only 3).
Field-collected samples	Fish swabs were collected in ethanol and transported with ice packs until they arrived to the lab where they were maintained at -20C until the time of the analyses.
Ethics oversight	Sampling was carried out under license ICMBio/SISBIO<br>57145-1/2017 and approved by Swansea University Ethics Committee reference SU-Ethics-Student-250717/245.<br>The sampling took place in 2017, before Brazil ratified the Nagoya protocol in 2021, however we ensured to follow Brazilian national laws, get prior informed consent from the authorities to carry out the sapling through our local partners and shared benefits fairly and equitably.

Note that full information on the approval of the study protocol must also be provided in the manuscript.

Plants

Seed stocks	NA
Novel plant genotypes	NA
Authentication	NA
